# Reproductive Health Literacy and Knowledge Among Female Refugees: A Scoping Review of Measurement Methodologies and Effect on Health Behavior

**DOI:** 10.3390/ijerph22071121

**Published:** 2025-07-16

**Authors:** Kimberly W. Tseng, Henna Mohabbat, Anne Adachi, Angela Calaguas, Amardeep Kaur, Nabeala Salem, Zahra Goliaei

**Affiliations:** 1 Department of Public Health, Touro University California, 1310 Club Dr, Vallejo, CA 94592, USA; zgoliaei@touro.edu; 2 College of Medicine, Touro University California, 1310 Club Dr, Vallejo, CA 94592, USA; hmohabba2@student.touro.edu (H.M.); aadachi@student.touro.edu (A.A.); acalagua@student.touro.edu (A.C.); akaur18@student.touro.edu (A.K.); nsalem3@student.touro.edu (N.S.)

**Keywords:** refugee, post-resettlement, reproductive health literacy, health behavior, decision-making

## Abstract

Reproductive health literacy (RHL) is essential to women’s ability to make informed reproductive health (RH) decisions and is a key determinant of RH outcomes. Resettled refugee women often experience poorer RH outcomes, yet there is limited research on their RHL and its influence on RH decision-making. This scoping review aims to (1) to evaluate existing methods for measuring RHL among resettled refugee women and (2) to characterize the relationship between RHL, RH decision-making, behavior, and outcomes among refugee women residing in high-income countries. A search of peer-reviewed literature published in English found limited direct measurement of RHL. Measurement methods were primarily qualitative or based on unvalidated survey instruments, limiting comparability and generalizability. The current methodologies do not adequately capture RH knowledge or RHL proficiency. A range of additional factors were found to influence RH decision-making and behavior, supporting the need for a means to accurately measure RHL. Further quantitative research is needed to clarify the extent to which RHL and knowledge influence RH behavior and outcomes. The development of a culturally relevant, validated RHL instrument that integrates knowledge and contextual influences would support healthcare providers and public health agents in serving and designing effective interventions for refugee women post-resettlement.

## 1. Introduction

Health literacy, a predictor of health behavior and health service utilization, is defined as the “knowledge, motivation and competences to access, understand, appraise, and apply health information in order to make judgments and take decisions in everyday life concerning healthcare, disease prevention and health promotion to maintain or improve quality of life during the life course” [1]. Low health literacy is associated with decreased healthcare service utilization, poorer health, and higher mortality [2]. With respect to reproductive health, reproductive health literacy greatly influences a woman’s ability to make informed decisions related to contraception, pregnancy, abortion, and other sexual and reproductive health concerns [3]. In studies evaluating the reproductive health literacy of women in London, higher reproductive health literacy was associated with the utilization of low-failure contraceptive methods and lower rates of unplanned pregnancy [4]. In contrast, low sexual and reproductive health literacy corresponded with a 44% increase in pregnancy prevalence and higher pregnancy frequency [5,6]. These findings suggest a potential influence of reproductive health literacy, to varying degrees, on individual reproductive health behavior and outcomes.

**Figure 1:**
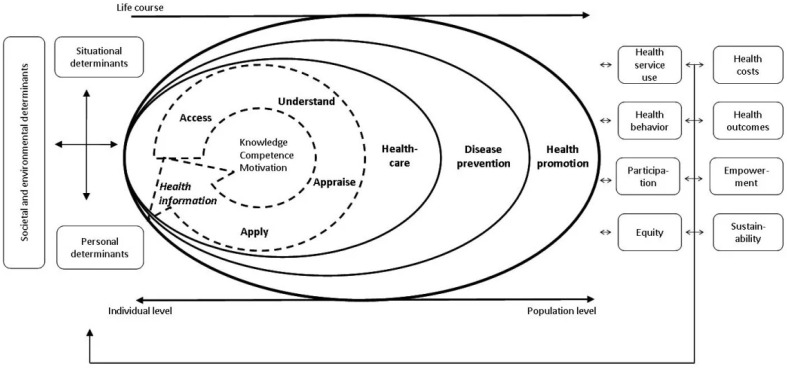
Sørensen’s integrated model of health literacy. Reproduced from Sørensen et al. (2012), BMC Public Health, 12(1), 80. Licensed under CC BY 2.0 [1].

Comparably, reproductive health knowledge may also function as a predictor of RH behavior. General health knowledge is a precursor to, a component of, and a product of health literacy [7,8]. Together with other determinants of health literacy (such as personal, situational, and societal/environmental determinants, as seen in Sørensen’s [1] integrated model of health literacy depicted in Figure 1), health knowledge has a significant influence on health literacy and thereby health-related beliefs and decision-making. This model is also observable in reproductive health domains, where greater reproductive health knowledge is associated with improved oral contraceptive adherence, while limited knowledge is linked to reduced utilization [9,10,11,12].

The degree to which each of these factors influences the health behavior specifically of refugee women residing in their non-native countries is unknown. Given the significant role societal and cultural influences play in forming attitudes, beliefs, and behavior related to reproductive health, women from refugee backgrounds likely experience barriers and facilitators of reproductive health literacy and behavior to different degrees than non-refugee populations, such as limited access to social support and effective, culturally relevant care in their resettled country [13]. Disparities in reproductive health suggest unaddressed needs in this population. In the U.S., women from refugee backgrounds were reported to have significantly lower prevalence of mammograms, with 86% of refugee women over the age of 40 having never received a mammogram compared to 33% of non-refugee women [14]. A similar pattern is seen with Pap smear testing. When compared to non-refugee migrants in Australia, women from humanitarian source countries were more likely to have late first pregnancy care visits, post-term birth, and poor or no pregnancy care attendance than women from non-humanitarian source countries [15]. A review of antenatal and prenatal female refugee outcomes in multiple high-income countries found higher rates of miscarriage, stillbirth, and perinatal mortality, despite lower rates of tobacco and drug usage during pregnancy [16]. Clarifying the role of reproductive health literacy and knowledge in shaping reproductive health outcomes of female refugee communities is critical to developing effective and evidence-based interventions post-resettlement.

There are currently an estimated 42.7 million refugees globally, 50% of whom are women, and 27% of whom are hosted in high-income countries [17]. This population has tripled in the last decade and continues to grow exponentially; however, despite having outpaced the growth of the world’s population, while simultaneously at higher risk for poorer reproductive health outcomes, relatively little investigation has been conducted to examine the relationship between reproductive health literacy, knowledge, behavior, and outcomes of refugee women post-resettlement [18]. For the purposes of this review, we use the term refugee women to refer to women from refugee or asylum-seeking backgrounds. According to the UNHCR, refugees and asylum seekers are individuals who have fled their home country due to persecution, conflict, or violence [19]. Compared to other immigrant populations, refugee and asylum-seeking women often face distinct structural, cultural, and psychological barriers to reproductive healthcare after resettlement, shaped by the unique circumstances of their migration [20]. While refugee and asylum-seeking women differ in legal status and may face distinct entitlements or access to services depending on their host country’s policies, both groups were included under a single term due to their shared experiences of forced migration and the structural, cultural, and psychological barriers they commonly encounter when seeking reproductive healthcare post-arrival. This scoping review examined the existing literature to evaluate the current capacity to measure the reproductive health literacy and knowledge of women from refugee backgrounds post-resettlement in high-income countries.

## 2. Methods

### 2.1. Study Design

This review was conducted in accordance with the methodological framework outlined by Arksey and O’Malley [21], and it adheres to the Preferred Reporting Items for Systematic reviews and Meta-Analyses extension for Scoping Reviews (PRISMA-ScR) checklist, provided in Appendix A [22]. A formal review protocol was not developed. Due to the emerging nature of the subject, the heterogeneity of the population, the inclusion of both quantitative and qualitative studies, and the exploratory nature of the research question, a scoping review approach was most appropriate [23].

### 2.2. Study Aim

To provide a comprehensive picture of reproductive health literacy with respect to the resettled refugee population, this scoping review aims to (1) identify and summarize the measures, tools, or indicators that have been used to evaluate reproductive health literacy or knowledge of women from refugee backgrounds residing in high-income countries and (2) examine any association between reproductive health literacy or knowledge and reproductive health behavior, decision-making, or outcomes. The findings are intended to guide healthcare providers (HCPs) and public health agencies in addressing reproductive health disparities in refugee communities and inform future research agendas aimed at better understanding the needs of resettled refugee women.

### 2.3. Definitions and Inclusion Criteria

This review included studies that met the following criteria: (1) comprised original, peer-reviewed articles employing qualitative, quantitative, or mixed-methods designs, published in or after 2013; (2) focused on refugee populations residing in high-income countries; (3) examined or measured participants’ literacy, knowledge, or awareness of any sexual or reproductive health (SRH) topic; and (4) were available in English. Studies were excluded if they did not specifically measure or assess participant knowledge or literacy. While RH outcomes and behavior were of interest, studies that solely examined outcomes, behavior, or decision-making without measuring RH knowledge or literacy were excluded, as our primary interest in outcomes was their relationship to RH literacy and knowledge.

The inclusion criteria were informed based on the World Health Organization’s definition of reproductive health, which encompasses a broad range of topics, including abortion, antenatal care, family planning, gender-based violence, HIV, sexually transmitted infections (STIs), maternal mortality, menstruation and gynecological health, and obstetric care [24]. In addition, studies were included if they examined refugee women’s awareness, knowledge, or literacy regarding any aspect of sexual or reproductive health.

### 2.4. Data Sources and Search Strategy

The initial search was conducted in December 2023 and updated in September 2024 and May 2025 across three databases: Google Scholar, PUBMED, and Scopus. These databases were selected for their comprehensive coverage of medical, public health, and interdisciplinary literature. Medline, Embase, or Global Health were not included due to institutional access constraints and substantial content overlap with the selected databases.

The search was performed in three iterative rounds to refine search terms and inclusion/exclusion criteria. The final search strategy combined terms to capture relevant studies: (“refugee” OR “refugee women” OR “refugees” OR “resettlement”) AND (“reproductive health” OR “pregnancy” OR “contraception” OR “autonomy” OR “reproductive behavior” OR “reproductive decision”) AND (“health literacy” OR “reproductive literacy” OR “health knowledge” OR “reproductive knowledge”). Although the inclusion criteria encompassed a broad range of reproductive health topics, the search strategy employed a focused set of representative terms to ensure search efficiency and reduce the retrieval of irrelevant records.

### 2.5. Study Selection

**Figure 2:**
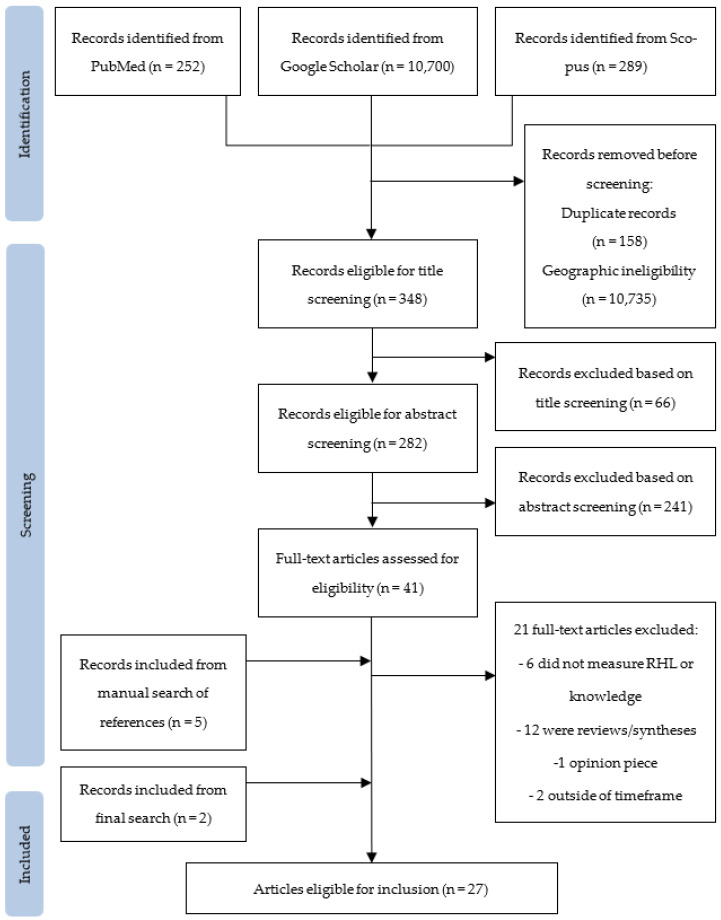
PRISMA flow diagram.

All retrieved titles were merged, and duplicate records were removed. The screening process was then performed in two-steps. In the first phase, titles and abstracts were independently screened by five team members (H.M., A.A., A.C., A.K., and N.S.) using predefined inclusion criteria, then reviewed by the first and last authors, who were not involved in the initial screening (K.T. and Z.G.). In the second stage, full-text articles were reviewed by all authors to determine final eligibility. Discrepancies were resolved through group consensus. The initial database search yielded 348 articles. After removing duplicates and conducting the screening process, 41 articles were retained. Of these, 20 articles met all eligibility criteria following full-text review. Citation screening identified 10 additional articles, of which 5 were included in the final analysis. No additional eligible articles were identified during a 2024 search update (performed by H.M.). Two additional articles were identified during a 2025 search update (performed by K.T.). A summary of the search and selection process is presented in Figure 2.

### 2.6. Data Extraction and Analysis

The first author and the last author (K.T. and Z.G.) identified key variables based on this study’s aims. These included study design, study population, geographic location, reproductive health domain, measurement of reproductive health knowledge/literacy, tools used to assess reproductive health knowledge/literacy, reported reproductive health behaviors and outcomes, indicators of these reproductive health behavior/outcomes, and any reported association between reproductive health knowledge/literacy and reproductive health behaviors, outcomes, or decision-making.

The first and last authors (K.T. and Z.G.) independently extracted all relevant data using a standardized form designed for this review. The extracted data was then cross-checked and reviewed to ensure completeness and consistency. Preliminary findings were compiled in a data extraction table. A summary of the extracted data is presented in Table 1.

## 3. Results

### 3.1. Study Characteristics

From 348 articles, 27 studies met the inclusion criteria. Among these articles, there were 14 qualitative, nine quantitative, and four mixed-method studies. Two articles, Kaneoka et al. [39] and Henry et al. [37], qualitatively assessed reproductive health literacy with no formal measurement. Rauf et al. [49] created and validated a reproductive health literacy scale for Afghan refugees with a focus on cervical cancer care, family planning, and maternal health/postpartum care. The remaining articles assessed knowledge of specific reproductive health domains, rather than reproductive health literacy. Family planning knowledge was the most common reproductive health domain addressed, with 13 studies measuring knowledge regarding contraception, birth spacing, and other methods of birth control. Nine articles assessed cervical cancer knowledge, one article assessed gender-based violence, three articles assessed HIV knowledge, two articles assessed maternal care knowledge, one article assessed menstruation and gynecological health knowledge, and five articles assessed knowledge regarding STIs (this domain was always measured in conjunction with others). No studies evaluated knowledge of abortion. From these 27 studies, 15 studies directly assessed the relationship between reproductive health knowledge and decision-making, behavior, or outcomes, of which nine were qualitative, five were quantitative, and one was mixed-method. No studies evaluated the relationship between reproductive health literacy and decision-making/behavior/outcomes.

With regard to geographic distribution, 12 of the studies took place in the U.S., and nine were in Australia. Less common geographical settings included Canada (one), Greece (one), Germany (two), Norway (one), Scotland (one), and Turkey (one).

### 3.2. Reproductive Health Literacy and Knowledge: Measurement Methods

#### 3.2.1. Reproductive Health Literacy

Only one article, Rauf et al., sought to measure reproductive health literacy across domains [49]. This study’s objective was specifically to create a reproductive health literacy measurement tool for refugee women that could be used to assess reproductive health literacy trainings for Afghan refugees. The primary domains of focus were family planning, maternal health, and cervical cancer prevention. However, the scale also included questions on general health literacy (adapted from HLS-EU-Q6) and digital health literacy (adapted from eHEALS). Reproductive health domain-specific questions were adapted from the Cervical Cancer Literacy Assessment Tool (C-CLAT) and the Refugee Reproductive Health Network (ReproNet) postpartum literacy scale. These scales were selected due to previous validations for the population addressed in the study.

#### 3.2.2. Cervical Cancer and Screening

Nine articles measured knowledge adequacy of cervical cancer and screening methods. Of the measurement methods, there were five questionnaire-based measures [28,29,31,36,48], four focus groups [27,36,41,43], one semi-structured interview [30], and one in-depth interview [43]. All questionnaires and focus group/interview guides were developed explicitly for their individual studies with the exception of the survey used in Dalla et al. [31] and Rauf et al. [49], which utilized adaptations of the Cervical Cancer Awareness Measure (Cervical CAM) and C-CLAT, respectively.

#### 3.2.3. Contraception and Family Planning

A total of 13 studies measured knowledge of topics relating to family planning and contraception. To measure knowledge, three of these studies utilized focus groups [25,50,51], four utilized in-depth interviews [35,40,46,47], two utilized semi-structured interviews [33,39], and one utilized group concept-mapping [44]. In these articles, all interview guides were designed specifically for each study. Three studies utilized questionnaire-based indicators, with the tools used in Dean et al. [32] and Napier-Raman et al. [45] adapted from the National Survey of Australian Secondary Students and Sexual Health. The third questionnaire-based study created their survey specifically for their investigation [38].

#### 3.2.4. HIV

Three articles assessed female refugees’ knowledge of HIV and HIV screening, all of which utilized questionnaires to quantitatively determine participants’ level of knowledge about HIV and screening practices. Two studies utilized indicators adapted from existing validated surveys: Dean et al. [32] used the questionnaire from the National Survey of Australian Secondary Students and Sexual Health (NSASSSH), and Feresu et al. [34] adapted their survey from questionnaires used by WHO, the UN Program on HIV/AIDS, CDC, and other similar studies. The remaining article developed a survey specifically for the study by consulting academic experts to develop the survey questions [26].

#### 3.2.5. Maternal Health and Pregnancy

A total of three studies measured knowledge about maternal care, including ante- and postnatal care and pregnancy. Two studies evaluated knowledge adequacy through interviews [37,42]. In addition to knowledge assessment by interview, Madeira et al. [42] also administered a questionnaire, which was created for a previous similar study, but not based on a validated tool. This is similar to Rauf et al. [49], which assessed knowledge through a previously created postpartum literacy scale.

#### 3.2.6. Other Domains

The remainder of the reproductive health domains—gender-based violence, menstruation/gynecological health and STIs—were never the primary focus of the studies and were consistently measured only alongside other topics. Knowledge of gender-based violence was qualitatively measured using group concept mapping in Napier-Raman et al. [44]. Knowledge in regard to menstruation and STIs was assessed in Metusela et al. [43] via focus groups and in-depth interviews. Other studies evaluating STI knowledge also evaluated family planning knowledge, all of which were qualitative [39,44,46], except for Dean et al. [32].

In total, only five of 27 reviewed studies (Dalla et al. [31], Dean et al. [32], Feresu et al. [34], Napier-Raman et al., 2025 [45], and Rauf et al. [49]) utilized sets of questions directly or adapted from validated measurement tools. An overview of the indicators and tools used in each study is presented in Table 2.

### 3.3. Reproductive Health Literacy and Knowledge: Relation to Behavior, Decision-Making, and Outcomes

#### 3.3.1. Cervical Cancer and Screening

All nine studies examining cervical cancer among refugee women reported limited knowledge of the disease, as well as of appropriate screening and prevention practices. Across multiple studies, reproductive health knowledge was strongly associated with health behaviors, such as Pap smear uptake, cervical cancer screening, and HPV vaccination—particularly when combined with enabling factors such as full-time employment, marriage, having children, or recent contact with a healthcare provider [27,28,30]. In contrast, low levels of knowledge were consistently linked to low screening rates, as seen in communities of refugees from Burma and Bhutan in the U.S. [41].

Participants across studies identified that both formal and informal health information played a critical role in developing self-efficacy and the confidence to make independent health decisions. Where knowledge was limited, misinformation and emotional barriers often emerged. These included inaccurate beliefs—for example, that HPV vaccines cause cancer or that cervical screening threatens virginity—as well as feelings of fear, embarrassment, and discomfort, particularly when interacting with male healthcare providers [28,30,41,43].

However, increasing awareness alone did not always lead to increased health behavior. In Anaman et al. [28], invitation letters designed to raise awareness of cervical cancer screening failed to improve screening rates. Self-initiated screenings remained nearly ten times lower among refugee women than non-refugees (2.2% vs. 21.0%), reflecting broader gaps in reproductive health literacy. In contrast, Ornelas et al. [48] demonstrated that targeted educational interventions could be more effective. Among Nepali–Bhutanese women, watching educational videos significantly improved awareness of cervical cancer testing (from 58% to 100%, *p* < 0.001), awareness of the Pap smear (from 45% to 100%, *p* < 0.001), and intention to screen (from 40% to 80%, *p* < 0.001). Among Karen–Burmese women, however, the results were more limited, with significant improvement observed only in Pap smear awareness (*p* = 0.008), and no change in screening intention.

#### 3.3.2. Contraception and Family Planning

This review found wide variation in both knowledge of contraception and contraceptive uptake across studies. In general, refugee and asylum-seeking women demonstrated lower levels of knowledge and contraceptive use than native-born populations. In Germany, women who perceived themselves as sexually educated were more likely to use contraception; however, knowledge level did not influence their preference for traditional versus modern methods, suggesting potential gaps in understanding contraceptive efficacy [38]. Similarly, among African Australian teenage mothers in Australia, post-resettlement, awareness increased through informal channels such as peers and media, but substantial gaps remained—particularly regarding mechanisms of action—which led to continued belief in myths and misinformation [46]. Similar trends were observed among Afghan refugees in Turkey, who exhibited low awareness of contraceptive options and lacked access to formal family planning education both before and after resettlement [40].

In contrast, Congolese refugee women in the U.S. demonstrated good knowledge of common contraceptive methods such as pills and injections but reported less awareness about long-acting methods such as IUDs and implants, particularly regarding side effects [50]. Somali Bantu refugee women in the U.S. were found to have a broad and accurate understanding of birth control options, despite limited formal education or literacy [25]. However, their contraceptive decisions were driven more by sociocultural influences than by knowledge. This was also observed in refugee youth in Australia: despite having higher levels of contraceptive knowledge than males, female refugee youth experienced higher rates of sexual coercion, STIs, and unplanned pregnancy, possibly due to gender norms that place the burden of reproductive responsibility on women [45].

Sociocultural norms and religious beliefs were key factors shaping attitudes and behaviors regarding contraception and family planning. In multiple contexts, stigma, cultural taboos, and traditional gender roles were reported as barriers to seeking reproductive health information and using contraception [33,39]. Among Somali immigrant women in Oslo, religious beliefs and poor access to reproductive health information fostered misconceptions and skepticism about contraceptives [35]. Similarly, Sudanese refugee youth in Australia reported cultural stigma as a major barrier to condom negotiation [32]. Refugee women in Glasgow described how cultural and religious norms limited their openness to discussing reproductive health and seeking care [39].

Across several studies, family and social networks were central to reproductive health decisions. In Metusela et al. [43], participants described negotiating contraception use with husbands, parents, and in-laws. Among Somali Bantu and Congolese women in the U.S., decision-making was often driven by male partners, regardless of the woman’s knowledge level [25,50]. Similarly, Afghan refugees in Turkey relied heavily on the influence of family and friends for reproductive health information and decisions [40]. In Australia, parental sexual health literacy and attitudes significantly shaped daughters’ knowledge and contraceptive behaviors [47].

In addition to sociocultural and informational barriers, structural challenges also limited contraceptive use. Refugee women in Glasgow reported that low reproductive health knowledge and barriers to information severely constrained their ability to make informed reproductive decisions [39]. For Congolese women in the U.S., meeting basic survival needs—such as securing housing, food, and employment—took priority over seeking reproductive healthcare, regardless of their awareness or intentions [50]. Among Somali refugees in the U.S., challenges such as lack of social support and emotional stress further hindered contraception uptake [51].

#### 3.3.3. HIV

A community of African refugees in the U.S. had low levels of HIV knowledge and high levels of stigma. However, higher knowledge was not associated with lower levels of stigma, and testing rates were relatively high, with 49.5% of study participants reporting a history of screening [26]. Similarly, among Sudanese Queenslanders, Dean et al. [32] found low and inaccurate knowledge regarding HIV and STIs despite a desire for more information. Low knowledge was also associated with higher rates of sexual risk behavior. In the midwestern U.S., Somali Bantu and Sudanese immigrant women with less than primary school education were more likely to have inaccurate knowledge about the HIV/AIDS test and safe sex practices, but relatively good knowledge regarding HIV transmission (mean score, 10.1/14) [34].

#### 3.3.4. Maternal Health and Pregnancy

With regard to maternal health and pregnancy, knowledge was shown to influence care utilization and care seeking behavior. In Germany, the knowledge level of Arabic-speaking refugee women was found to be insufficient to recognize the need for healthcare in a timely manner during pregnancy and childbirth, resulting in delayed care [37]. An experimental study with Somali refugee and immigrant women showed that a group prenatal care model, Hooyo, increased knowledge about multiple pregnancy and prenatal topics (safe exercise in pregnancy (*p* = 0.02), breastfeeding (*p* = 0.04), hospital experience (*p* = 0.02) and stress management (*p* = 0.03)) and also resulted in increased post-intervention engagement in care [42].

#### 3.3.5. Other Domains

A large qualitative study of migrant and refugee women from Afghanistan, Iraq, Somalia, South Sudan, Sudan, India, Sri Lanka, and South America—resettled in Sydney, Australia, and Vancouver, Canada—found widespread self-reported gaps in knowledge across multiple sexual and reproductive health domains, including menstruation, fertility, contraception, cervical screening, HPV vaccination, and STIs [43]. Similarly, migrant and refugee youth in Australia reported feeling inadequately educated about reproductive health topics, particularly in areas such as healthy relationships, boundaries, and consent. However, decision-making was not driven by knowledge alone; interpersonal consequences were also cited as major influences on behavior [44].

Low levels of STI knowledge were also reported among Australian refugee youth in another study, which found a link between limited knowledge and increased sexual risk behaviors [32].

## 4. Discussion

### 4.1. Measurement of Reproductive Health Literacy and Knowledge

Our ability to measure reproductive health literacy of refugee women post-resettlement is limited. As such, most studies evaluated reproductive health knowledge and its influence on decision-making and behavior. While reproductive health knowledge influences reproductive health literacy, the two are not synonymous; reproductive health literacy encompasses other enabling factors, such as cognitive, emotional, and practical skills, which make it a more comprehensive predictor of reproductive health behavior compared to reproductive health knowledge alone [2,52]. In addition, the studies included in our review relied predominantly on qualitative methods. While qualitative approaches allow for rich exploration of the many factors shaping reproductive health literacy and behavior, they limit the ability to quantify and evaluate the strength of associations between reproductive health literacy, knowledge, behavior, and health outcomes. Only five studies in this review used validated tools to measure reproductive health knowledge, while four others relied on study-specific instruments with limited standardization. Furthermore, only two studies employed experimental designs, reducing the reliability and generalizability of conclusions about the relationship between reproductive health knowledge and reproductive health behaviors.

Most of the validated tools identified in our review—Cervical CAM, NSASSSH, and a WHO questionnaire—were developed for specific reproductive health domains and non-refugee populations, limiting their applicability across broader reproductive health topics or refugee contexts. The only exception to this was the ReproNet reproductive health literacy scale, which adapted the HLS-EU-Q6 (general health literacy), eHEALS (digital literacy), reproductive health literacy (C-CLAT), and ReproNet postpartum literacy scale, and was validated for Afghan refugees in the U.S. [24]. The ReproNet scale represents an important step toward refugee-specific measurement but places relatively limited emphasis on structural access barriers, which may significantly shape the reproductive health experiences of refugees.

Refugee women may hold coherent and contextually appropriate reproductive health beliefs that differ from the biomedical frameworks dominant in high-income countries. When measurement tools fail to account for these belief systems, individuals may be viewed as having low health knowledge or literacy, not due to lack of understanding or capacity, but because their interpretations of health information conflict with prevailing norms of their new environment [53]. This further underscores the need for a culturally responsive and validated RHL tool specifically designed for refugee populations post-resettlement. Ideally, a comprehensive reproductive health literacy scale would measure across multiple reproductive health domains and account for the unique cultural, structural, and interpersonal factors influencing reproductive health literacy and decision-making among refugee women. Based on the studies reviewed, relevant factors include factual knowledge and comprehension; awareness of and ability to access reproductive health services and resources; effective communication with providers; decision-making autonomy; sources of reproductive health information; norms, beliefs, and perceptions; and the ability to meet basic daily needs. Existing quantitative reproductive health literacy tools (e.g., SHELA, SHLS, SRHL-Q for Lao adolescents, and the Reproductive Health Literacy Questionnaire for Chinese Unmarried Youth) have been validated only in specific national contexts [54,55,56,57,58] and remain underutilized in refugee populations post-resettlement. Instead, most research continues to depend on knowledge screenings and qualitative data, resulting in inconsistent and often insufficient measurement of reproductive health literacy in these communities.

### 4.2. The Influence of Reproductive Health Knowledge on Decision-Making, Behavior, and Outcomes

Several studies in this review found that increased reproductive health knowledge was generally associated with improved decision-making, greater engagement with reproductive health services, and more positive health outcomes. Conversely, limited knowledge was linked to lower screening rates and reduced uptake of health-promoting behaviors. In the two experimental studies reviewed [42,48], participants who received reproductive health education—via group prenatal classes or cervical cancer videos—showed increases in healthcare utilization, engagement, and behavioral intention. These findings align with broader evidence linking general health knowledge to positive health behaviors [2].

However, while knowledge played a role, many studies highlighted the importance of other enabling factors that shaped reproductive health decisions and behaviors among refugee women. Several studies found that perceived risk had a stronger influence on behavior than knowledge alone, suggesting that subjective and sociocultural factors significantly impact reproductive health decision-making [28,30,33,37,44].

Key individual and interpersonal factors included predisposing experiences (e.g., trauma, marital status, past healthcare encounters [37,41]), stigma (particularly related to HIV and family planning [33,36,41,47]), and emotional barriers (such as fear, mistrust, and embarrassment [30,51]). Sociocultural influences were also central: religious and gender norms, partner approval, cultural expectations, and reliance on informal sources of information were frequently cited as influential [25,27,30,32,33,34,35,39,40,41,42,43,44,45,46,47,50,51].

At the structural level, barriers such as transportation difficulties [36,43], lack of reproductive health education during clinical encounters [27,30], limited access to female providers [30], poor continuity of care [36,37], challenges navigating health systems [31,33,36,41], and financial constraints [35,36,41,46,50,51] were frequently reported.

The degree of influence of reproductive health knowledge appears to vary across reproductive health domains. Studies focusing on cervical cancer and screening behaviors were more likely to identify knowledge as a primary factor of service utilization and behavior, possibly due to the presence of structured screening programs and less ingrained social norms, compared to domains such as family planning or HIV. This suggests the need for more nuanced, domain-specific research to better understand the how reproductive health knowledge influences reproductive health behavior and outcomes. This is particularly important given that current interventions for resettled refugee women often prioritize knowledge improvement as the primary measurement of success, rather than tangible health outcomes [59]. Future studies should quantitatively assess how reproductive health knowledge influences decision-making and behavior within each reproductive health domain with the objective of creating a model that functions in the context of refugee and migrant communities. Interventions focused on culturally sensitive topics such as family planning, HIV, and maternal health should use a culture-centered framework; consider non-education/awareness-based strategies; and, where appropriate, integrate traditional practices to increase acceptability and effectiveness [40].

### 4.3. Promoting Reproductive Health Literacy and Knowledge

Our findings support a high frequency of inadequate reproductive health knowledge and literacy among refugee populations, though, as expected, this is not consistent across all reproductive health domains or populations. This aligns with a broader body of research that documents persistent gaps in reproductive health literacy and knowledge in LMICs and humanitarian settings [60,61,62]. We found two exceptions in our review. Agbemenu et al. [25] found adequate knowledge regarding birth control options among a Somali Bantu refugee community in Buffalo, New York, and Royer et al. [50] observed relatively high levels of knowledge and awareness concerning birth spacing methods among Congolese women in a metropolitan area in the western U.S. Notably, both studies focused specifically on contraception, and both concluded that adequate knowledge did not necessarily translate into behavioral change.

All other studies in our review reported low levels of knowledge or misinformation, particularly in relation to HIV [26,32,34], cervical cancer [27,28,29,30,31,36,41,43,48], and maternal health [37,42]. The comparatively higher levels of contraception knowledge may reflect the longstanding prioritization of family planning in global reproductive health funding, which has often framed contraception as a tool for economic development and health equity in low- or middle-income countries (LMICs) [63,64]. Family planning also remains a central focus of reproductive health interventions in humanitarian settings [61,65]. Future research should examine how global reproductive health education initiatives are distributed across reproductive health domains—both within and beyond humanitarian contexts—and how that distribution affects knowledge and behavior following resettlement. Programs pursuing global reproductive justice should expand their educational scope beyond contraception to address broader reproductive health concerns within resettled refugee communities.

Several studies in this review identified actionable opportunities to promote reproductive health knowledge directly within refugee populations. Some participants demonstrated a strong desire for more reproductive health information and took initiative to seek it out, often through internet sources [32,37,39,44]. Participants also cited web-based multimedia (including videos and digital reading materials) as helpful resources. Peer educators, particularly those who share the same cultural and religious backgrounds, and group care models for pre- and postnatal care were also reported as acceptable and effective strategies for increasing reproductive health knowledge [42,43]. While promising, these strategies require further evaluation, especially in resettled refugee contexts. Additionally, expanding reproductive health education to include digital health tools such as mHealth and mobile learning could be impactful, as both have demonstrated effectiveness in delivering health education to refugee populations in other domains [66,67].

HCPs were consistently identified as trusted sources of reproductive health information—sometimes preferred even over family or community leaders [27,32,39]. This positions HCPs as potential key actors in reducing reproductive health knowledge gaps and promoting reproductive health literacy. Strategies to support this role include conducting comprehensive patient histories to uncover migration- and trauma-related experiences; adopting a culture-centered approach to education; promoting shared goals in patient–provider relationships; integrating education into routine care; and improving cultural competence through provider training. Additionally, HCPs may benefit from using whole-family approaches to education and decision-making when appropriate. Structural interventions can also enhance reproductive health knowledge acquisition—such as diversifying the healthcare workforce and ensuring financial coverage for professional medical interpreters, who play a crucial role in patient comprehension and communication [27,37,39].

### 4.4. Limitations

This review is subject to several limitations that affect the generalizability and interpretability of its findings. First, the lack of uniformity in how reproductive health literacy and knowledge are measured across studies presents a challenge for this review. The absence of a validated, standardized reproductive health literacy measurement tool limits the comparability of study results and hinders our ability to draw strong conclusions about the relationship between reproductive health literacy, reproductive health knowledge, and health behaviors across different populations and contexts. The diverse and inconsistent methods used to assess knowledge and literacy—ranging from study-specific survey instruments to qualitative interviews—make it difficult to evaluate the strength of associations or aggregate findings across studies.

Second, generalizability is also affected by the wide heterogeneity of refugee populations included in the reviewed studies. Differences in cultural background, language, migration experiences, host country health systems, and legal status reduce the extent to which findings can be applied to refugee and asylum-seeking populations more broadly. While this diversity provides important insight into the varying contexts of reproductive health decision-making, it also complicates synthesis and limits the ability to give focused recommendations. Additionally, because our search was limited to studies that explicitly identified participants as refugees or asylum seekers, relevant studies that assessed reproductive health literacy or knowledge among refugee populations but categorized them more broadly under general immigrant groups were excluded [68]. However, given the unique experiences of migration and resettlement that often differ significantly from the experience of non-refugee populations, narrowing the scope of our review may strengthen the relevance and specificity of our findings to the refugee experience. Additionally, assessing refugees and asylum seekers as a single group may obscure important differences in service eligibility and legal protections, particularly in countries where access to health services for asylum seekers is significantly restricted. While the decision to combine these categories was informed by their overlapping experiences of forced displacement and marginalization, we acknowledge that their legal distinctions may influence health behavior and access in ways that this review does not fully capture. Future studies should consider disaggregating findings by legal status where possible to better understand the effect of policy-related barriers on reproductive health literacy.

Third, this review may have been influenced by publication bias. Only peer-reviewed literature was included; therefore, relevant findings from gray literature, unpublished reports, or community-level evaluations may have been omitted. Although comprehensive search strategies were used—including multiple database queries and reference mining—some eligible studies may still have been missed. Furthermore, the review was limited to studies published in English, which may have excluded relevant research conducted in non-English-speaking countries, particularly those hosting large refugee populations.

Finally, as a scoping review focused on thematic synthesis, this review does not include a formal quality appraisal of included studies. While this approach allows for the mapping of broad themes and gaps in the literature, it limits the ability to comment on the rigor or methodological quality of the individual studies reviewed.

## 5. Conclusions

This review aimed to provide a comprehensive overview of our current ability to measure the reproductive health literacy and knowledge of refugee women in high-income countries and to identify gaps in understanding the relationship between reproductive health literacy, knowledge, behavior, decision-making, and outcomes. Our findings reveal that the current literature is limited in its use of validated, standardized tools to measure reproductive health literacy, with most studies focusing instead on reproductive health knowledge, mostly using inconsistent and study-specific instruments. While reproductive health knowledge is an important component of reproductive health literacy, our analysis suggests that independently, it is an insufficient predictor of reproductive health behaviors or outcomes, particularly in populations facing complex sociocultural, interpersonal, and structural barriers.

To strengthen future research and improve reproductive health outcomes of female refugees, there is a critical need for validated, culturally relevant tools that measure reproductive health literacy across multiple reproductive health domains. Such tools would support providers and policymakers in assessing needs, tailoring interventions, and evaluating impact. In addition, a reproductive health literacy scale would improve our ability to assess the effectiveness of health promotion interventions. Future interventions should not only address knowledge gaps but also work to facilitate communication, foster trust in healthcare systems, and improve access to culturally and linguistically appropriate resources. Addressing the broader determinants of reproductive health behavior in refugee populations will require integrated, equity-focused strategies that go beyond addressing reproductive health knowledge through traditional health education models.

## Figures and Tables

**Table 1:** Characteristics of the studies included in the review.

N	Author	Year	Country	Population (*n*)	Design	Findings
1	Agbemenu et al. [25]	2018	USA	Somali Bantu refugee women (30)	Qualitative	Accurate and high levels of knowledge on birth control options did not increase contraceptive uptake.
2	Agbemenu et al. [26]	2022	USA	African refugee women (101)	Quantitative	Study population had overall low levels of knowledge. Accurate knowledge did not override stigma.
3	Allen et al. [27]	2018	USA	Somali Bantu refugee women with >1 child (31)	Qualitative	Low knowledge about HPV was associated with low HPV vaccination rates.
4	Anaman et al. [28]	2017	Australia	African refugee (144) and non-refugee (110) women	Quantitative	Low health literacy and low levels of knowledge regarding cervical cancer and screening were associated with low Pap smear uptake.
5	Anaman et al. [29]	2018	Australia	African refugee (144) and non-refugee (110) women	Quantitative	Refugees in the study population had significantly lower levels of knowledge about cervical cancer and Pap smear screening.
6	Anaman-Torgbor et al. [30]	2017	Australia	African refugee (10) and non-refugee (9) women	Qualitative	Low knowledge was identified as a barrier to cervical cancer screening participation.
7	Dalla et al. [31]	2022	Greece	Syrian refugee women (176)	Quantitative	Study population had extreme low levels of knowledge regarding cervical cancer, screening methods, and HPV vaccination, assessed using cervical CAM.
8	Dean et al. [32]	2017	Australia	Sudanese refugee-background youth, aged 16–24 (80 female and 149 male)	Quantitative	Low levels of STI and HIV knowledge were associated with higher sexual risk behavior. Knowledge was measured using NSASSSH.
9	Dhar et al. [33]	2017	USA	Bhutanese refugee female youth (14)	Qualitative	Study population had low levels of knowledge across RH domains.
10	Feresu et al. [34]	2013	USA	Sudanese (86) and Somali Bantu (14) immigrant women from predominantly refugee community	Mixed methods	Knowledge on different aspects of HIV (transmission, protection, testing, etc.) was generally low and associated with low rate of condom usage.
11	Gele et al. [35]	2020	Norway	Somali immigrant women (21)	Qualitative	Low levels of knowledge regarding contraceptives was associated with nonuse.
12	Haworth et al. [36]	2014	USA	Bhutanese refugee women (69)	Mixed methods	Limited knowledge was identified as a barrier to Pap test utilization. History of Pap smear was associated with increased knowledge.
13	Henry et al. [37]	2020	Germany	Iraqi, Syrian, and Palestinian refugee women (12)	Qualitative	Low health literacy and knowledge regarding maternal care was associated with delays in seeking care.
14	Inci et al. [38]	2020	Germany	Refugee women from various countries (307)	Quantitative	History of sexual education was associated with contraceptive usage, but not associated with preference for more effective contraceptive methods.
15	Kaneoka et al. [39]	2020	Scotland	Asylum-seeking and refugee women from various countries (14)	Qualitative	RH literacy was low in the study population, which was identified as a barrier to RH decision-making.
16	Kuru Alici and Ogüncer [40]	2024	Turkey	Afghan refugee women (20)	Qualitative	Low and inaccurate knowledge was not associated with nonuse of contraceptives.
17	Lor et al. [41]	2018	USA	Refugee women from Burma (31) and Bhutan (27)	Qualitative	Low cervical cancer knowledge was associated with low rates of screening. Health information was identified as a facilitator of health behavior and independent health decision-making.
18	Madeira et al. [42]	2019	USA	Somali women from a predominantly refugee community (21)	Mixed methods	Participation in group prenatal care was associated with increased knowledge. Increased knowledge was associated with increased engagement in prenatal care.
19	Metusela et al. [43]	2017	Australia, Canada	Migrant and refugee women from Afghanistan (35), Iraq (27), Somalia (38), South Sudan (11), Sudan (20), India (9), Sri Lanka (12), and South America (17)	Qualitative	Study population had inadequate knowledge across multiple RH domains. Inaccurate knowledge was a barrier to RH behavior.
20	Napier-Raman et al. [44]	2024	Australia	Migrant and refugee youth, aged 16–26 (42 female and 13 male) from various countries (68)	Mixed methods	Study participants had lack of RH knowledge and education. Relational factors were more influential in the decision-making process than knowledge.
21	Napier-Raman et al. [45]	2025	Australia	Migrant and refugee youth, aged 16–26 (74 female and 32 male) from various countries (107)	Quantitative	Females had greater knowledge and awareness of contraceptive methods than males, but misconceptions persisted in both genders. Contraceptive utilization was not different between genders. Women had higher rates of sexual coercion, STIs, and unplanned pregnancy.
22	Ngum Chi Watts et al. [46]	2014	Australia	Refugee teenagers and women from Sudan (10), Liberia (3), Ethiopia (1), Burundi (1), and Sierra Leone (1) with h/o teenage pregnancy (16)	Qualitative	Low knowledge surrounding contraceptives was identified as a deterrent to contraceptive uptake.
23	Ngum Chi Watts et al. [47]	2015	Australia	Refugee teenagers and women from Sudan (10), Liberia (3), Ethiopia (1), Burundi (1), and Sierra Leone (1) with h/o teenage pregnancy (16)	Qualitative	Low and inaccurate knowledge was associated with nonuse of contraceptives.
24	Ornelas et al. [48]	2017	USA	Karen-Burmese (20) and Nepali–Bhutanese (20) refugee women	Quantitative	Increased knowledge after watching cervical cancer educational videos was not consistently associated with increased intention to pursue Pap screening.
25	Rauf et al. [49]	2025	USA	Afghan refugees (184), specifically Dari (67), Arabic (53), and Pashto (64) speakers	Quantitative	Reproductive health literacy scale made of HLS-EU-Q6, eHEALS, C-CLAT, and SHELA showed good inter-item reliability for this population.
26	Royer et al. [50]	2019	USA	Somali (41) and Congolese (25) refugee women	Qualitative	High levels of knowledge regarding available methods of contraception was not associated with contraceptive usage.
27	Zhang et al. [51]	2020	USA	Somali refugee women of reproductive age (53)	Qualitative	Inaccurate knowledge was a barrier to contraceptive uptake.

**Table 2:** Indicators of knowledge/literacy of reproductive health domains. Specific tools listed if used [25,26,27,28,29,30,31,32,33,34,35,36,37,38,39,40,41,42,43,44,45,46,47,48,49,50,51].

		Agbemenu et al., 2018 [25]	Agbemenu et al., 2022 [26]	Allen et al., 2018 [27]	Anaman et al., 2017 [28]	Anaman et al., 2018 [28]	Anaman-Torgbor et al., 2017 [30]	Dalla et al., 2022 [31]	Dean et al., 2017 [32]	Dhar et al., 2017 [33]	Feresu et al., 2013 [34]	Gele et al., 2020 [35]	Haworth et al., 2014 [36]	Henry et al., 2020 [37]	Inci et al., 2020 [38]	Kaneoka et al., 2020 [39]	Kuru Alici & Ogüncer, 2024 [40]	Lor et al., 2018 [41]	Madeira et al., 2019 [42]	Metusela et al., 2017 [43]	Napier-Raman et al., 2024 [44]	Napier-Raman et al., 2025 [45]	Ngum Chi Watts et al., 2014 [46]	Ngum Chi Watts et al., 2015 [47]	Ornelas et al., 2017 [48]	Rauf et al., 2025 [49]	Royer et al., 2019 [50]	Zhang et al., 2020 [51]
Domain	Abortion																											
	Cervical Cancer			X	X	X	X	CAM					X					X		X					X	ReproNet **		
	Family Planning	X							NSASSSH	X		X			X	X	X			X	X	NSASSSH	X	X		ReproNet **	X	
	Gender-Based Violence																				X							
	HIV		X						X		WHO *																	
	Maternal Health and Obstetric Care													X					X							ReproNet **		
	Menstruation and Gynecological Health																			X								
	STIs								X							X				X	X			X				

* Tool not specified. ** ReproNet RHL scale: adaptation of HLS-EU-Q6 (general health literacy), eHEALS (digital literacy), C-CLAT, and ReproNet postpartum literacy scale.

## Data Availability

The data presented in this study are available on request from the corresponding author.

## Abbreviations

The following abbreviations are used in this manuscript:

HCP	Healthcare provider
LMIC	Low- or middle-income country
RH	Reproductive health
RHL	Reproductive health literacy
SRH	Sexual and reproductive health

## Appendix A

**Table A1:** PRISMA-ScR checklist.

Section	Item	PRISMA-ScR Checklist Item	Reported on Page #
Title	1	Identify the report as a scoping review.	1
Abstract			
Structured summary	2	Provide a structured summary that includes (as applicable): background, objectives, eligibility criteria, sources of evidence, charting methods, results, and conclusions that relate to the review questions and objectives.	1
Introduction			
Rationale	3	Describe the rationale for the review in the context of what is already known. Explain why the review questions/objectives lend themselves to a scoping review approach.	1–3
Objectives	4	Provide an explicit statement of the questions and objectives being addressed with reference to their key elements (e.g., population or participants, concepts, and context) or other relevant key elements used to conceptualize the review questions and/or objectives.	3
Methods			
Protocol and registration	5	Indicate whether a review protocol exists; state if and where it can be accessed (e.g., a Web address); and if available, provide registration information, including the registration number.	3
Eligibility criteria	6	Specify characteristics of the sources of evidence used as eligibility criteria (e.g., years considered, language, and publication status), and provide a rationale.	3
Information sources *	7	Describe all information sources in the search (e.g., databases with dates of coverage and contact with authors to identify additional sources), as well as the date the most recent search was executed.	3
Search	8	Present the full electronic search strategy for at least 1 database, including any limits used, such that it could be repeated.	3–4
Selection of sources of evidence †	9	State the process for selecting sources of evidence (i.e., screening and eligibility) included in the scoping review.	4
Data charting process ‡	10	Describe the methods of charting data from the included sources of evidence (e.g., calibrated forms or forms that have been tested by the team before their use, and whether data charting was done independently or in duplicate) and any processes for obtaining and confirming data from investigators.	4
Data items	11	List and define all variables for which data were sought and any assumptions and simplifications made.	5, Table 1 and Table 2
Critical appraisal of individual sources of evidence §	12	If done, provide a rationale for conducting a critical appraisal of included sources of evidence; describe the methods used and how this information was used in any data synthesis (if appropriate).	NA
Synthesis of results	13	Describe the methods of handling and summarizing the data that were charted.	4
Results			
Selection of sources of evidence	14	Give numbers of sources of evidence screened, assessed for eligibility, and included in the review, with reasons for exclusions at each stage, ideally using a flow diagram.	8
Characteristics of sources of evidence	15	For each source of evidence, present characteristics for which data were charted and provide the citations.	Table 1 and Table 2
Critical appraisal within sources of evidence	16	If done, present data on critical appraisal of included sources of evidence (see item 12).	NA
Results of individual sources of evidence	17	For each included source of evidence, present the relevant data that were charted that relate to the review questions and objectives.	Table 1 and Table 2
Synthesis of results	18	Summarize and/or present the charting results as they relate to the review questions and objectives.	9–12
Discussion			
Summary of evidence	19	Summarize the main results (including an overview of concepts, themes, and types of evidence available), link to the review questions and objectives, and consider the relevance to key groups.	12–15
Limitations	20	Discuss the limitations of the scoping review process.	15–16
Conclusions	21	Provide a general interpretation of the results with respect to the review questions and objectives, as well as potential implications and/or next steps.	16
Funding	22	Describe sources of funding for the included sources of evidence, as well as sources of funding for the scoping review. Describe the role of the funders of the scoping review.	18

JBI = Joanna Briggs Institute; PRISMA-ScR = Preferred Reporting Items for Systematic reviews and Meta-Analyses extension for Scoping Reviews. * Where sources of evidence (see second footnote) are compiled from, such as bibliographic databases, social media platforms, and Web sites. † A more inclusive/heterogeneous term used to account for the different types of evidence or data sources (e.g., quantitative and/or qualitative research, expert opinion, and policy documents) that may be eligible in a scoping review as opposed to only studies. This is not to be confused with information sources (see first footnote). ‡ The frameworks by Arksey and O’Malley (6) and Levac and colleagues (7) and the JBI guidance (4, 5) refer to the process of data extraction in a scoping review as data charting. § The process of systematically examining research evidence to assess its validity, results, and relevance before using it to inform a decision. This term is used for items 12 and 19 instead of “risk of bias” (which is more applicable to systematic reviews of interventions) to include and acknowledge the various sources of evidence that may be used in a scoping review (e.g., quantitative and/or qualitative research, expert opinion, and policy document).

## References

[1] Sørensen K., Van Den Broucke S., Fullam J., Doyle G., Pelikan J., Slonska Z., Brand H., (HLS-EU) Consortium Health Literacy Project European (2012). Health Literacy and Public Health: A Systematic Review and Integration of Definitions and Models. BMC Public Health.

[2] Berkman N.D., Sheridan S.L., Donahue K.E., Halpern D.J., Crotty K. (2011). Low Health Literacy and Health Outcomes: An Updated Systematic Review. Ann. Intern. Med..

[3] Corneliess C., Gray K., Kidwell Drake J., Namagembe A., Stout A., Cover J. (2022). Education as an Enabler, Not a Requirement: Ensuring Access to Self-Care Options for All. Sex. Reprod. Health Matters.

[4] Ghavami B., Sohrabi Z., RaisiDehkordi Z., Mohammadi F. (2024). Relationship between Reproductive Health Literacy and Components of Healthy Fertility in Women of the Reproductive Age. J. Educ. Health Promot..

[5] Dongarwar D., Salihu H.M. (2019). Influence of Sexual and Reproductive Health Literacy on Single and Recurrent Adolescent Pregnancy in Latin America. J. Pediatr. Adolesc. Gynecol..

[6] Bahrampour B., Shahali S., Lamyian M., Rasekhi A. (2024). Sexual Health Literacy among Rural Women in Southern Iran. Sci. Rep..

[7] Von Wagner C., Steptoe A., Wolf M.S., Wardle J. (2009). Health Literacy and Health Actions: A Review and a Framework From Health Psychology. Health Educ. Behav..

[8] Freedman D.A., Bess K.D., Tucker H.A., Boyd D.L., Tuchman A.M., Wallston K.A. (2009). Public Health Literacy Defined. Am. J. Prev. Med..

[9] Alomair N., Alageel S., Davies N., Bailey J.V. (2020). Factors Influencing Sexual and Reproductive Health of Muslim Women: A Systematic Review. Reprod. Health.

[10] Hall K.S., Castaño P.M., Westhoff C.L. (2014). The Influence of Oral Contraceptive Knowledge on Oral Contraceptive Continuation Among Young Women. J. Women’s Health.

[11] Kilfoyle K.A., Vitko M., O’Conor R., Bailey S.C. (2016). Health Literacy and Women’s Reproductive Health: A Systematic Review. J. Women’s Health.

[12] Tomaszewski D., Aronson B.D., Kading M., Morisky D. (2017). Relationship between Self-Efficacy and Patient Knowledge on Adherence to Oral Contraceptives Using the Morisky Medication Adherence Scale (MMAS-8). Reprod. Health.

[13] Morris M.D., Popper S.T., Rodwell T.C., Brodine S.K., Brouwer K.C. (2009). Healthcare Barriers of Refugees Post-Resettlement. J. Community Health.

[14] Barnes D.M., Harrison C.L. (2004). Refugee Women’s Reproductive Health in Early Resettlement. J. Obstet. Gynecol. Neonatal Nurs..

[15] Gibson-Helm M.E., Teede H.J., Cheng I., Block A.A., Knight M., East C.E., Wallace E.M., Boyle J.A. (2015). Maternal Health and Pregnancy Outcomes Comparing Migrant Women Born in Humanitarian and Nonhumanitarian Source Countries: A Retrospective, Observational Study. Birth.

[16] Sturrock S., Williams E., Greenough A. (2021). Antenatal and Perinatal Outcomes of Refugees in High Income Countries. J. Perinat. Med..

[17] United Nations High Commissioner for Refugees (2025). Global Trends: Forced Displacement in 2024. https://www.unhcr.org/global-trends-report-2024.

[18] Davidson N., Hammarberg K., Romero L., Fisher J. (2022). Access to Preventive Sexual and Reproductive Health Care for Women from Refugee-like Backgrounds: A Systematic Review. BMC Public Health.

[19] United Nations High Commissioner for Refugees (2025). Refugee Data Finder: Methodology. https://www.unhcr.org/refugee-statistics/methodology.

[20] Stirling-Cameron E., Almukhaini S., Dol J., DuPlessis B.J., Stone K., Aston M., Goldenberg S.M. (2024). Access and use of sexual and reproductive health services among asylum-seeking and refugee women in high-income countries: A scoping review. PLoS ONE.

[21] Arksey H., O’Malley L. (2005). Scoping Studies: Towards a Methodological Framework. Int. J. Soc. Res. Methodol..

[22] Tricco A.C., Lillie E., Zarin W., O’Brien K.K., Colquhoun H., Levac D., Moher D., Peters M.D.J., Horsley T., Weeks L. (2018). PRISMA Extension for Scoping Reviews (PRISMA-ScR): Checklist and Explanation. Ann. Intern. Med..

[23] Peters M.D.J., Marnie C., Tricco A.C., Pollock D., Munn Z., Alexander L., McInerney P., Godfrey C.M., Khalil H. (2020). Updated Methodological Guidance for the Conduct of Scoping Reviews. JBI Evid. Synth..

[24] World Health Organization (2006). Reproductive Health Indicators: Guidelines for Their Generation, Interpretation and Analysis for Global Monitoring. https://iris.who.int/bitstream/handle/10665/43185/924156315X_eng.pdf.

[25] Agbemenu K., Volpe E.M., Dyer E. (2018). Reproductive Health Decision-making among US -dwelling Somali Bantu Refugee Women: A Qualitative Study. J. Clin. Nurs..

[26] Agbemenu K., Aidoo-Frimpong G., Auerbach S., Jafri A. (2022). HIV Attitudes and Beliefs in U.S.-Based African Refugee Women. Ethn. Health.

[27] Allen E.M., Lee H.Y., Pratt R., Vang H., Desai J.R., Dube A., Lightfoot E. (2018). Facilitators and Barriers of Cervical Cancer Screening and Human Papilloma Virus Vaccination Among Somali Refugee Women in the United States: A Qualitative Analysis. J. Transcult. Nurs..

[28] Anaman J.A., Correa-Velez I., King J. (2017). A Survey of Cervical Screening among Refugee and Non-refugee African Immigrant Women in Brisbane, Australia. Health Prom. J. Aust..

[29] Anaman J.A., Correa-Velez I., King J. (2018). Knowledge Adequacy on Cervical Cancer Among African Refugee and Non-Refugee Women in Brisbane, Australia. J. Canc. Educ..

[30] Anaman-Torgbor J.A., King J., Correa-Velez I. (2017). Barriers and Facilitators of Cervical Cancer Screening Practices among African Immigrant Women Living in Brisbane, Australia. Eur. J. Oncol. Nurs..

[31] Dalla V., Panagiotopoulou E.-K., Deltsidou A., Kalogeropoulou M., Kostagiolas P., Niakas D., Labiris G. (2022). Level of Awareness Regarding Cervical Cancer Among Female Syrian Refugees in Greece. J. Canc. Educ..

[32] Dean J., Mitchell M., Stewart D., Debattista J. (2017). Sexual Health Knowledge and Behaviour of Young Sudanese Queenslanders: A Cross-Sectional Study. Sex. Health.

[33] Dhar C.P., Kaflay D., Dowshen N., Miller V.A., Ginsburg K.R., Barg F.K., Yun K. (2017). Attitudes and Beliefs Pertaining to Sexual and Reproductive Health Among Unmarried, Female Bhutanese Refugee Youth in Philadelphia. J. Adolesc. Health.

[34] Feresu S., Smith L. (2013). Knowledge, Attitudes, and Beliefs about HIV/AIDS of Sudanese and Bantu Somali Immigrant Women Living in Omaha, Nebraska. OJPM.

[35] Gele A.A., Musse F.K., Shrestha M., Qureshi S. (2020). Barriers and Facilitators to Contraceptive Use among Somali Immigrant Women in Oslo: A Qualitative Study. PLoS ONE.

[36] Haworth R.J., Margalit R., Ross C., Nepal T., Soliman A.S. (2014). Knowledge, Attitudes, and Practices for Cervical Cancer Screening Among the Bhutanese Refugee Community in Omaha, Nebraska. J. Community Health.

[37] Henry J., Beruf C., Fischer T. (2020). Access to Health Care for Pregnant Arabic-Speaking Refugee Women and Mothers in Germany. Qual. Health Res..

[38] Inci M.G., Kutschke N., Nasser S., Alavi S., Abels I., Kurmeyer C., Sehouli J. (2020). Unmet Family Planning Needs among Female Refugees and Asylum Seekers in Germany—Is Free Access to Family Planning Services Enough? Results of a Cross-Sectional Study. Reprod. Health.

[39] Kaneoka M., Spence W. (2020). The Cultural Context of Sexual and Reproductive Health Support: An Exploration of Sexual and Reproductive Health Literacy among Female Asylum Seekers and Refugees in Glasgow. IJMHSC.

[40] Kuru Alici N., Ogüncer A. (2024). Knowledge, Beliefs, and Cultural Practices of Sexual and Reproductive Health Among Afghan Refugee Women in Türkiye. J. Transcult. Nurs..

[41] Lor B., Ornelas I.J., Magarati M., Do H.H., Zhang Y., Jackson J.C., Taylor V.M. (2018). We Should Know Ourselves: Burmese and Bhutanese Refugee Women’s Perspectives on Cervical Cancer Screening. J. Health Care Poor Underserved.

[42] Madeira A.D., Rangen C.M., Avery M.D. (2019). Design and Implementation of a Group Prenatal Care Model for Somali Women at a Low-Resource Health Clinic. Nurs. Women’s Health.

[43] Metusela C., Ussher J., Perz J., Hawkey A., Morrow M., Narchal R., Estoesta J., Monteiro M. (2017). “In My Culture, We Don’t Know Anything About That”: Sexual and Reproductive Health of Migrant and Refugee Women. Int. J. Behav. Med..

[44] Napier-Raman S., Hossain S.Z., Mpofu E., Lee M.-J., Liamputtong P., Dune T. (2024). Sexual and Reproductive Health and Rights Decision-Making among Australian Migrant and Refugee Youth: A Group Concept Mapping Study. Cult. Health Sex..

[45] Napier-Raman S., Bidewell J., Hossain S.Z., Mpofu E., Lee M.-J., Liamputtong P., Dune T. (2025). Migrant and Refugee Youth’s Sexual and Reproductive Health and Rights: A Gender Comparison of Knowledge, Behaviour, and Experiences. Sex. Cult..

[46] Ngum Chi Watts M.C., Liamputtong P., Carolan M. (2014). Contraception Knowledge and Attitudes: Truths and Myths among African Australian Teenage Mothers in Greater Melbourne, Australia. J. Clin. Nurs..

[47] Ngum Chi Watts M.C., McMichael C., Liamputtong P. (2015). Factors Influencing Contraception Awareness and Use: The Experiences of Young African Australian Mothers. J. Refug. Stud..

[48] Ornelas I.J., Ho K., Jackson J.C., Moo-Young J., Le A., Do H.H., Lor B., Magarati M., Zhang Y., Taylor V.M. (2017). Results From a Pilot Video Intervention to Increase Cervical Cancer Screening in Refugee Women. Health Educ. Behav..

[49] Rauf M., Goliaei Z., Machta L., Chang J., Thiel De Bocanegra H. (2025). Reproductive Health Literacy Scale: A Tool to Measure the Effectiveness of Health Literacy Training. Reprod. Health.

[50] Royer P.A., Olson L.M., Jackson B., Weber L.S., Gawron L., Sanders J.N., Turok D.K. (2019). Family Planning Knowledge, Attitudes, and Practices Among Somali and Congolese Refugee Women After Resettlement to the United States. Qual. Health Res..

[51] Zhang Y., McCoy E.E., Scego R., Phillips W., Godfrey E. (2020). A Qualitative Exploration of Somali Refugee Women’s Experiences with Family Planning in the U.S. J. Immigr. Minor. Health.

[52] Alhussaini N.W.Z., Elshaikh U., Abdulrashid K., Elashie S., Hamad N.A., Al-Jayyousi G.F. (2025). Sexual and Reproductive Health Literacy of Higher Education Students: A Scoping Review of Determinants, Screening Tools, and Effective Interventions. Glob. Health Action.

[53] Ma X., Yang Y., Wei Q., Jiang H., Shi H. (2021). Development and Validation of the Reproductive Health Literacy Questionnaire for Chinese Unmarried Youth. Reprod. Health.

[54] Dowling A., Enticott J., Russell G. (2017). Measuring Self-Rated Health Status Among Resettled Adult Refugee Populations to Inform Practice and Policy—A Scoping Review. BMC Health Serv. Res..

[55] Maasoumi R., Tavousi M., Zarei F. (2019). Development and Psychometric Properties of Sexual Health Literacy for Adults (SHELA) Questionnaire. Hayat J. Sch. Nurs. Midwifery.

[56] Panahi R., Dehghankar L., Amjadian M. (2022). Investigating the Structural Validity and Reliability of the Sexual Health Literacy for Adults (SHELA) Questionnaire among a Sample of Women in Qazvin, Iran. BMC Women’s Health.

[57] Rashidi K., Watson P., Farahani H., Chesli R.R., Abiri F.A. (2023). Developing and Validating the Sexual Health Literacy Scale in an Iranian Adult Sample. Humanit. Soc. Sci. Commun..

[58] Vongxay V., Thongmixay S., Stoltenborg L., Inthapanyo A., Sychareun V., Chaleunvong K., Rombout Essink D. (2022). Validation of the Questionnaire on Sexual and Reproductive Health Literacy for Adolescents Age 15 to 19 Years in Lao People’s Democratic Republic. HLRP Health Lit. Res. Pract..

[59] Bitterfeld L., Ozkaynak M., Denton A.H., Normeshie C.A., Valdez R.S., Sharif N., Caldwell P.A., Hauck F.R. (2025). Interventions to Improve Health Among Refugees in the United States: A Systematic Review. J. Community Health.

[60] Amanu A., Birhanu Z., Godesso A. (2023). Sexual and Reproductive Health Literacy among Young People in Sub-Saharan Africa: Evidence Synthesis and Implications. Glob. Health Action.

[61] Desrosiers A., Betancourt T., Kergoat Y., Servilli C., Say L., Kobeissi L. (2020). A Systematic Review of Sexual and Reproductive Health Interventions for Young People in Humanitarian and Lower-and-Middle-Income Country Settings. BMC Public Health.

[62] Soeiro R.E., De Siqueira Guida J.P., da-Costa-Santos J., Costa M.L. (2023). Sexual and Reproductive Health (SRH) Needs for Forcibly Displaced Adolescent Girls and Young Women (10–24 Years Old) in Humanitarian Settings: A Mixed-Methods Systematic Review. Reprod. Health.

[63] Nandagiri R. (2021). What’s so Troubling about ‘Voluntary’ Family Planning Anyway? A Feminist Perspective. Popul. Stud..

[64] Senderowicz L., Valley T. (2023). Fertility Has Been Framed: Why Family Planning Is Not a Silver Bullet for Sustainable Development. St. Comp. Int. Dev..

[65] Jennings L., George A.S., Jacobs T., Blanchet K., Singh N.S. (2019). A Forgotten Group during Humanitarian Crises: A Systematic Review of Sexual and Reproductive Health Interventions for Young People Including Adolescents in Humanitarian Settings. Confl. Health.

[66] El-Haj-Mohamad R., Nohr L., Niemeyer H., Böttche M., Knaevelsrud C. (2023). Smartphone-Delivered Mental Health Care Interventions for Refugees: A Systematic Review of the Literature. Camb. Prism. Glob. Ment. Health.

[67] Meyer C.L., Surmeli A., Hoeflin Hana C., Narla N.P. (2022). Perceptions on a Mobile Health Intervention to Improve Maternal Child Health for Syrian Refugees in Turkey: Opportunities and Challenges for End-User Acceptability. Front. Public Health.

[68] Vazquez Corona M., Hazfiarini A., Vaughan C., Block K., Bohren M.A. (2024). Participatory Health Research With Women From Refugee, Asylum-Seeker, and Migrant Backgrounds Living in High-Income Countries: A Scoping Review. Int. J. Qual. Methods.

